# Possible ability of bovine follicular fluid to attract migrating bull spermatozoa

**DOI:** 10.1002/rmb2.12025

**Published:** 2017-04-04

**Authors:** MD Anisuzzaman Mondal, Yuji Takagi, Shoji A. Baba, Koh‐ichi Hamano

**Affiliations:** ^1^ Faculty of Agriculture Shinshu University Kamiina Japan; ^2^ Faculty of Science Ochanomizu University Tokyo Japan

**Keywords:** bull, chemotaxis, follicular fluid, motility, sperm

## Abstract

**Aim:**

To examine the potential of bovine follicular fluid (BFF) to attract bull spermatozoa.

**Methods:**

The ability of the BFF to attract bull sperm was evaluated by observing changes in sperm migration after being placed in a cross‐column chamber. The movement parameters of the heads and flagella of the sperm that were attracted to the BFF were analyzed by using the Computer Assisted Sperm Analysis system.

**Results:**

It was observed that 61.6% of the bull sperm migrated toward the BFF when the BFF was used at a concentration of 0.1%, but 67.2% of the sperm did not migrate toward the BFF at a concentration of 10%. Relatively larger numbers of both precapacitated and postcapacitated bull sperm migrated toward the BFF (0.1%). The ability of the 0.1% BFF to attract sperm probably affected both the normal artificial insemination (AI) fertility sperm and the poor AI fertility spermatozoa. The flagellar curvilinear ratio of the sperm winding to the 0.1% BFF was significantly higher than that of the prewinding sperm.

**Conclusion:**

These results could suggest that BFF potentially attracts bull sperm at a certain concentration, irrespective of the capacitation status of the sperm. Although the mechanism by which this attraction occurs remains unclear, these data imply that it could be related to BFF‐dependent changes in the sperm flagellar curvilinear ratio.

## 1 Introduction

Sperm chemotaxis has been confirmed in species that have been fertilized in vitro.^1^, ^2^ Mammalian sperm migrate in the female genital tract and fertilize in the ampulla of the uterine tube. To date, the sperm motility, migration ability, and capacitation, which are essential for fertilization, have been studied.^3^, ^4^, ^5^ In vivo sperm motility and migration are influenced strongly by the physiological state of contraction of, and fluid secretion in, the female genital tract. Based on a detailed kinetic analysis of migrated sperm in the female genital tract, it was found that chemotaxis,^1^ thermotaxis,^6^, ^7^ and rheotaxis^8^ are involved in sperm migration. In mammals, swine sperm chemotaxis toward the leukocytes^9^ and rabbit and human sperm chemotaxis toward the follicular fluid have been reported.^10^, ^11^ The follicular fluid includes the fluid that is secreted from the ovary and follicular cells and affects the physiological function and motility of the sperm. Sperm chemotaxis has been analyzed conventionally by using the needle method and the chamber method.^1^ In studies of the chemotactic movement of the sperm of marine invertebrates, the sperm trajectory has been examined mainly in vitro. Ascidian sperm swim in a straight line when they are exposed to a chemoattractant, and if the sperm should move away from the chemoattractant, they make sudden quick changes in direction to turn back toward the chemoattractant.^2^, ^12^ Calcium bursts are induced by the concentration of the chemoattractant: sperm‐activated and attracting factors triggers a sequence of flagellar responses that comprise quick turns in order to direct the sperm toward the eggs.^13^ There have been few studies on the motility mechanism of mammalian sperm chemotaxis. Although the trajectory of the sperm head has been examined,^10^, ^14^, ^15^, ^16^ there is little detailed analysis of the flagellar movement. Sperm motility can be analyzed objectively and exactly by using the Computer Assisted Sperm Analysis (CASA) system.

The aim of this study was to evaluate the ability of bovine follicular fluid (BFF) to attract sperm by observing the changes that occur in the sperm's migration and movement parameters in response to the BFF in a cross‐column chamber. Based on the obtained results, it is discussed whether bull spermatozoa have the potential to exhibit chemotaxis in response to the components of the BFF.

## 2 Materials and Methods

### 2.1 Media and chemicals

Brackett & Oliphant's (BO) medium,^17^ containing 1% bovine serum albumin (Wako, Tokyo, Japan) was used for the washing and dilution of the sperm and for the column construction. The sperm capacitation‐inducing medium, or HC, contained both 15 μg/mL of heparin (Mochida, Tokyo, Japan) and 5 mmol/L of caffeine (SIGMA‐Aldrich, St. Louis, MO, USA) with the BO medium. The BFF was obtained from bovine follicles (2–5 mm in diameter) that did not contain blood from the ovary that was obtained from a local slaughterhouse. All the fluids were pooled and centrifuged at 10 000*g *for 60 minutes at 4°C. Then, the supernatant was filtered and stored at −80°C until use. The frozen BFF was thawed and diluted with the BO medium to concentrations that ranged from 10% to 0.01% (v/v). The medium that was used for the washing and migration examination of the sperm was the BO medium, either with or without the BFF.

### 2.2 Semen

Frozen semen was generously donated by our association that provided the bull semen. This was collected from three Holstein Friesian bulls, referred to here as bulls A, B, and C, and then was frozen. Bulls A, B, and C showed different fertility levels, which were evaluated by using artificially inseminated cows. The conception rates of the cows that were inseminated with the frozen semen from bulls A, B, and C were 65% (324/495 heads), 57% (585/1029 heads), and 34% (95/277 heads), respectively. The evaluation of sperm motility and migration was conducted by using frozen semen from bull B and the relationship between the bull fertility levels and sperm migration was examined by using frozen semen from all three bulls.

### 2.3 Chamber preparation

**Figure 1:**
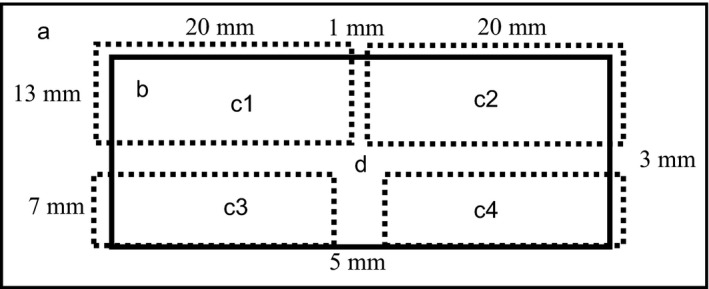
Analysis chamber used to evaluate the attraction of migrating sperm by bovine follicular fluid was constructed of 10 mm vertical (width: 5 mm) and 40 mm horizontal (width: 3 mm) cross‐type columns. The columns were connected with a 13 mm vertical part (width: 1 mm) to the object glass (A) by using 50 μm thick double‐sided tape (c1‐c4) and then adhered together with a cover glass (B) (22 mm×40 mm)

The attraction of the sperm was estimated by using manufactured analysis chambers with cross‐type columns. The cross‐type columns were constructed to be 7 mm long vertically (width: 5 mm) and 41 mm long horizontally (width: 3 mm). They were connected to the object glass (S2215; Matsunami, Kishiwada, Japan) with a 13 mm long vertical (width: 1 mm) part by using double‐sided tape that was 50 μm in thickness (No. 7046; Teraoka, Tokyo, Japan) and then adhered together with a 22 mm× 40 mm cover glass (No. 1; Matsunami) and filled with BO medium (Figure 1). Both ends of the wide column were covered with mineral oil after the sperm were introduced.

### 2.4 Sperm preparation

The frozen semen was thawed in a water bath at 38.5°C and diluted with an equal volume of BO medium. The diluted semen was washed three times by centrifugation at 150*g* for 5 minutes. The sperm concentration was adjusted to 1 × 10^7^/mL. Sperm pre‐incubation was conducted in a micro‐drop of BO medium or HC in a CO_2_ incubator (5% CO_2_/95% air) at 38.5°C for 4 hours. The sperm samples were used for the chlortetracycline (CTC) staining assay in order to evaluate the capacitation state. Staining was performed, as described previously.^18^ Briefly, the sperm suspension was treated with CTC solution for 30 seconds and mixed with paraformaldehyde in Tris buffer. The sperm suspension was placed on a glass slide and covered with a cover slip. The sperm were examined with a fluorescence microscope. The capacitated sperm (~50% of the examined sperm) were classified in the B pattern, consisting of a postacrosomal region with dark fluorescence and an intact acrosome with bright fluorescence.

### 2.5 Evaluation of the attraction of sperm and of sperm motility

A sperm suspension was obtained after washing or pre‐incubation in HC and the ratio of sperm motility was examined by using an examination plate (Sekisui, Tokyo, Japan) at 38.5°C. The motility was shown as a ratio of the total sperm (%). The sperm movements were recorded through a phase contrast microscope (BX50; Olympus, Tokyo, Japan) by using a SAG‐1050 Capture card (Ditect, Tokyo, Japan) that was connected to a XC‐HR300 charge coupled device (CCD) camera (Sony, Tokyo, Japan). For illumination, a power light‐emitting diode was mounted just under the condenser of the microscope. Its output was synchronized to the vertical drive signal of the CCD camera in order to produce a single flash of 100 μseconds duration per individual exposure. The recordings of the sperm motility were analyzed by taking photographs at 100 frames per second, with an exposure time of 1/1000 seconds under the phase contrast microscope. The sperm swimming paths and flagellar waveforms were analyzed by using the CASA system. The obtained images were recorded and analyzed by using the image analysis software, Bohboh (BohbohSoft, Tokyo, Japan).^19^, ^20^ Using the Bohboh software, the sperm head image and flagellar waveform could be tracked automatically and the swimming velocities, path curvatures, and flagellar curvature could be calculated from the tracking data, as follows: (i) straight‐line velocity (VSL), the distance between the first‐ and last‐tracked points divided by the time elapsed; (ii) curvilinear velocity (VCL), the sum of the distances between the adjacent points divided by the time elapsed; (iii) linearity (LIN), an index of the straightness of the path, given by VSL/VCL × 100; (iv) flagellar curvilinear ratio (FCR), an index of the curvilinearity of the flagella, calculated from the points corresponding to ~5 μm in flagellar length after 11 points are averaged; and (v) flagellar beat frequency (FBF), an index of vigor, given by the frequency with which the cell track crosses the smoothing path in either direction.

**Figure 2:**
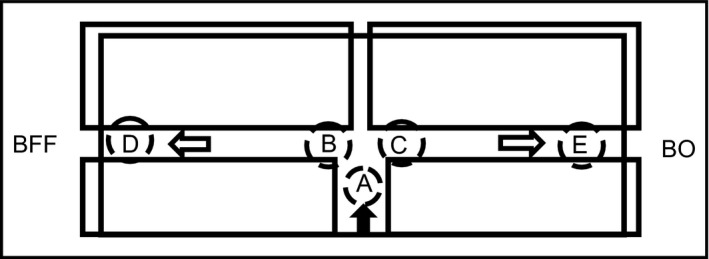
Evaluation of the motility and swimming paths of the migrating sperm that were subject to attraction in the cross‐type column. The analysis areas were selected for pre‐ (A), during (B,C), and post‐ (D,E) winding and migration at the ends of the vertical and horizontal columns of the chamber. BFF, bovine follicular fluid; BO, Brackett & Oliphant's medium

The attraction of the sperm by the BFF gradient was evaluated in the 41 mm long part of the cross‐type column (Figure 1). In the no‐BFF‐gradient condition, the BO medium (3 μL) was set at both ends of the wide column. In the BFF‐gradient condition, diluted BFF (diluted with BO medium from 10% to .01%) (3 μL) was set at one end of the wide column and BO medium (3 μL) was set at the other end (Figure 2). Then 10 minutes later, the sperm (3 μL) were introduced into the end of the vertical part (Figure 2; black arrow) of the cross‐type column and observed for 20 minutes. The attraction of the sperm was evaluated by measuring the number of sperm that arrived at the ends (Figure 2D,E) of both columns after they were immobilized by warming them to 60°C. The sperm's swimming paths and flagellar waveforms were analyzed at pre‐ (Figure 2A), during (Figure 2B,C), and post‐ (Figure 2D,E) winding and migration in the ends of the vertical and horizontal columns of the chamber.

### 2.6 Statistical analysis

Each experiment was replicated at least three times. The significance of the differences in the sperm motility and numbers of migrating sperm was determined with the Student's *t* test and the Mann‐Whitney *U*‐test. In some experiments, the significance was analyzed by using Dunnett's multiple test.

## 3 Results

### 3.1 Effect of the bovine follicular fluid concentration on the attraction of the sperm

The bull sperm migration to the different concentrations of BFF is shown in Table 1. The ratio of sperm migration to 0.1% BFF was 61.6%, which was significantly higher than that to the BO medium. The BFF has a weak attractant effect on sperm migration. The ratios of sperm migration to the BFF and 10% BFF were 26.3% and 32.8%, respectively, and were significantly lower than those to the BO medium. The ratios of sperm migration to 1% BFF and .01% BFF showed no remarkable difference from that to the BO medium.

**Table 1:** Effects of different concentrations of bovine follicular fluid (BFF) on the attraction of bull sperm

Chemoattractant	Migrating sperm	BFF	10% BFF	1% BFF	0.1% BFF	.01% BFF	BO medium
BO medium	No	188.9±15.7	235.8±19.4	176.1±15.3	105.7±11.4	129.5±12.8	131.6±14.9
	%	73.7±5.9a, A	67.2±5.1b, A	57.8±7.9	38.4±5.8c, A	44.9±7.6	46.4±7.1B
BFF	No	67.5±10.3	114.6±15.2	128.4±14.8	169.3±12.9	159.1 ±18.3	151.9±14.7
	%	26.3±5.8a, C	32.8±6.2b, C	42.2±8.1	61.6±6.0c, C	55.1±5.8	53.6±6.8D

Each value represents the mean±standard error of the mean of the results (the number and ratio of sperm) of 8‐10 experiments. Bull B semen was used in this experiment. The BFF (3 μL) and Brackett & Oliphant's (BO) medium (3 μL) were set at the opposite ends of the horizontal column of the manufactured analysis chamber. There were significant differences between the values marked with the same letters within the same columns, at *P*<.05.

### 3.2 Attraction of the precapacitated and the postcapacitated bull sperm

The motility and migration of the precapacitated and the postcapacitated frozen‐thawed bull sperm are shown in Table 2. The ratios of the migration of thte precapacitated and the postcapacitated bull sperm to 0.1% BFF were 62.1% and 59.6%, respectively, and were significantly higher than those to the BO medium. The motility, VCL, VSL, and LIN of the postcapacitated sperm tended to be lower than those of the precapacitated sperm. There was no association between the migration of the precapacitated and the postcapacitated sperm to the BFF and the kinetic parameters (VCL, VSL, and LIN).

**Table 2:** Motility and migration in the bovine follicular fluid (BFF) gradient of the bull sperm before and after the capacitation treatment

Sperm capacitation treatment^a^	Chemoattractant	Migrating sperm					
		No	%	Motility (%)	Curvilinear velocity (μm/s)	Straight‐line velocity (μm/s)	Linearity (%)
Before	BO medium	111.4±13.3	37.9±5.5a	51.4±6.1	138.4±19.5	78.1±8.3	56.5±6.2
	BFF	182.6±17.4	62.1±6.3a	52.3±7.5	171.3±21.9	93.5±10.2	54.4±5.3
After	BO medium	89.7±15.7	40.4±5.9b	40.6±4.9	105.7±14.8	48.6±5.1	45.7±4.5
	BFF	132.3±12.4	59.6±4.8b	43.7±5.1	123.6±17.1	59.4±6.3	48.0±5.1

a Sperm were treated in the capacitation media containing heparin and caffeine for 4 hours. Bull B semen was used in this experiment. The BFF (0.1%, 3 μL) and Brackett & Oliphant's (BO) medium (3 μL) were set at the opposite ends of the horizontal column of the manufactured analysis chamber. Each value represents the mean±standard error of the mean of the results of three‐to‐five experiments. There were significant differences between the values marked with the same letters within the same columns, at *P*<.05.

### 3.3 Bull fertility and the attraction of the sperm

The motility and migration of the frozen‐thawed sperm from the bulls with different fertility levels are shown in Table 3. The ratios of sperm migration to 0.1% BFF for bulls A, B, and C were 63.9%, 61.8%, and 59.2%, respectively, and were significantly higher than those to the BO medium. There was no remarkable difference in the motility or LIN of the sperm from bulls A, B, and C. The VCL and VSL of the migration of the sperm from bull A to the BFF were significantly higher than those of the migration of the sperm from bull C. No clear association was found between bull fertility and the motility parameters of the sperm that were attracted to the BFF.

**Table 3:** Motility and migration in the bovine follicular fluid (BFF) gradient of the sperm from the bulls with different fertility

Bull	Chemoattractant	Migrating sperm					
		No	%	Motility (%)	Curvilinear velocity (μm/s)	Straight‐line velocity (μm/s)	Linearity (%)
A	BO medium	116.6±13.5	36.1±4.4a	55.5±6.4	144.5±16.2	85.2±9.2	59.0±7.2
	BFF	206.4±19.4	63.9±6.8a	54.8±7.2	195.8±20.9d	109.4±11.3e	55.9±6.4
B	BO medium	112.7±10.3	38.2±5.6b	50.3±7.1	129.6±18.1	71.9±8.7	55.0±4.7
	BFF	182.3±20.3	61.8±6.1b	53.4±5.6	166.1±22.2	88.2±6.6	53.0±5.5
C	BO medium	112.2±14.1	40.8±4.5c	49.1±5.3	102.3±9.5	48.1±5.9	47.1±4.9
	BFF	162.8±15.2	59.2±5.3c	51.7±6.5	111.7±13.8d	52.7±7.1e	46.8±5.4

Fertility level of bulls A, B, and C, in terms of the conception ratios of the cows after artificial insemination, were 65%, 57%, and 34%, respectively. The BFF (0.1%, 3 μL) and Brackett & Oliphant's (BO) medium (3 μL) were set at the opposite ends of the horizontal column of the manufactured analysis chamber. Each value represents the mean±standard error of the mean of the results of three‐to‐five experiments. There were significant differences between the values marked with the same letters within the same columns, at *P*<.05.

### 3.4 Motility of the attracted migrating sperm

The motility levels of the frozen‐thawed sperm pre‐ (Figure 2A), during (Figure 2B,C), and post‐ (Figure 2D,E) winding and migration to 0.1% BFF or BO medium at both ends of the horizontal column of the chamber are shown in Table 4. The area (Figure 2A‐E) of the cross‐type column that was used for the sperm motility analysis is shown in Figure 2. From the analysis of the trajectory of the sperm that migrated to the BFF, it was determined that the sperm wind and migrate as “winding sperm;” that is, between the prewinding point (Figure 2A) and the during‐winding point (Figure 2B) was an angular area of 45°C, in which the BFF‐responsive sperm had a higher probability of being located. The number of times that the “winding sperm” engaged in winding was one or more. The sperm that migrated to the BO medium showed a major gentle‐arc trajectory or migrated straight in the vertical column and then along the horizontal column. The FCR of the sperm (Figure 2B) as they wound to the BFF (0.1%) was significantly higher than that of the prewinding sperm. The VCL and VSL of the prewinding sperm (Figure 2A) in the vertical column tended to be high, but there was no remarkable difference in the LIN or FBF of these sperm (Table 4). The VCL, VSL, and FBF of the sperm (Figure 2B) that were winding to the BFF tended to be higher than for the others, but the difference was not significant (Table 4).

**Table 4:** Kinetic parameters of the chemotactically winding bull sperm in the bovine follicular fluid (BFF) gradient

Chemoattractant	Winding trajectory	VCL (μm/s)	VSL (μm/s)	LIN (%)	FCR (rad/μm)	FBF (Hz)
Before	Prewinding	202.6±19.7	107.3±9.8	50.2±6.3	0.03±0.01a	10.1±1.8
BO medium	Winding	165.5±17.6	79.2±6.9	47.9±5.1	0.05±0.02a	12.5±2.3
	Postwinding	131.9±15.4	75.4±5.7	57.3±5.9	0.05±0.01a	11.3±2.3
BFF	Winding	189.6±14.9	97.8±8.8	51.3±6.2	0.09±0.02b	13.4±1.8
	Postwinding	169.3±17.3	91.2±8.7	53.3±5.7	0.06±0.02a,b	12.2±2.7

Each value represents the mean ± standard error of the mean of the sperm from three‐to‐five experiments. The BFF (0.1%, 3 μL) and Brackett & Oliphant's (BO) medium (3 μL) were set at the opposite ends of the horizontal column of the manufactured analysis chamber. There were significant differences between the values marked with different letters within the same column, at *P*<.05. FBF, flagellar beat frequency; FCR, flagellar curvilinear ratio; LIN, linearity; VCL, curvilinear velocity; VSL, straight‐line velocity.

## 4 Discussion

In the human,^16^, ^21^, ^22^, ^23^ rabbit,^10^ and mouse,^15^ sperm chemotaxis to the follicular fluid and egg‐related material has been observed, but the mechanism of sperm chemotaxis is not sufficiently understood. In this study, it was investigated whether bull sperm can be attracted to the BFF. Then, the relationships of this attraction with the motility and capacitation states of the attracted sperm and the influences of bull fertility on the sperm attraction were examined.

In the rabbit and human, sperm chemotaxis to follicular fluid has been characterized. The sperm showed the strongest chemotaxis toward 10^3^‐ or 10^4^‐fold‐diluted (0.1% or 0.01%) follicular fluid.^10^, ^11^ In this study, the responsiveness of bull sperm to the BFF at the different concentrations of between 0.01% and 100% was compared and it was found that 0.1% BFF elicited the strongest response. This suggests that BFF has the ability to attract sperm, like rabbit and human follicular fluids, and that the ability of the follicular fluid (from the cow, rabbit, and human) to attract sperm could be shown off by the proper dilution with a culture media. The BFF might enhance the VSL and VCL of bull sperm and then increase the sperm motility. The results of this study are supported by reports^14^, ^15^ that show that the materials that have been derived from oocytes enhance the average path velocity and VSL of human and mouse sperm.

Capacitated sperm induce hyperactivation. A couple of studies have reported that chemotaxis is valid for the selection of the sperm that can reach the oocytes and that only the capacitated sperm can exhibit chemotaxis.^10^, ^11^ It also has been suggested that hyperactivation is involved in the changes of motility direction of the chemotaxis‐exhibiting human sperm.^16^ Although the involvement of calcium ions in the regulation of capacitation and chemotaxis has been shown, the occurrence of hyperactivation was not noticed in ascidian sperm that showed calcium bursts.^13^ In this study, the attraction to the BFF was observed in both the precapacitated and postcapacitated bull sperm. Furthermore, hyperactivation rarely occurred in the postcapacitated bull sperm that migrated to the BFF. Thus, the relationships of chemotaxis with capacitation and hyperactivation are likely to be different among species and are still controversial. In the chemotactic examinations that were performed in the cross‐type column of this study, the percentages of sperm that migrated to the BFF were significantly higher than those of the sperm that migrated to the BO medium, implying the possible chemotaxis of bull sperm toward BFF. In addition, the ratios of the sperm that migrated to 0.1% BFF were not significantly affected by different bull fertility levels and different sperm capacitation states.

In this study, in order to elucidate the motility mechanism of the chemotactic sperm, the motility and kinetic trajectory of the head and flagella of the chemotactic winding sperm to the BFF were analyzed by using the CASA system. The VCL and VSL of the sperm that were winding to the BFF tended to be higher than those of the sperm that were winding to the BO medium. Therefore, it was speculated that the bull sperm that were attracted to the BFF showed an increased VSL and VCL and then increased their sperm motility. The FCR of the winding sperm that were attracted to the BFF was significantly higher than that of the other sperm. Therefore, it is speculated that the migrating bull sperm that were attracted to the BFF changed their direction of migration toward a chemoattractant by changing their FCR. It has been reported that the change in the swimming direction toward a chemoattractant is induced by the quick turning of the swimming path with asymmetric waveforms in ascidian sperm.^24^ In order to elucidate the mechanism by which chemotactic bull sperm change their swimming direction, studying the sperm of marine animals might not be suggestive. Probably, the flagellar movement mechanism that is involved in the change of direction toward a chemoattractant would be different in the sperm between marine animals and mammalian animals. However, the basic structures of the flagellar axoneme are conserved among these animals. Interestingly, a study reported the details of the sliding of dynein motor‐driven microtubules in the flagellar axoneme during the sperm chemotaxis of marine animals.^25^ In order to elucidate the swimming direction‐changing mechanism of chemotactic bull sperm, information on the dynamics of the structural elements of the sperm flagellar axoneme might be useful.

In order to elucidate the sperm fertilization mechanism, analyses of thermotaxis, rheotaxis, and hyperactivation, in addition to chemotaxis, are necessary. In this study, BFF was suggested to be involved both in the increase of the bull sperm's VSL and VCL and in the changing of the swimming direction toward the BFF by means of flagellar bending in the chemotactic sperm. The results of this study will contribute to the elucidation of the bull sperm motility and fertilization functions.

## Disclosures

*Conflict of interest*: The authors declare no conflict of interest. *Human and Animal Rights*: All institutional and national guidelines for the care and use of laboratory animals were followed.
